# Physicians' perceptions of patient participation and the involvement of family caregivers in the palliative care pathway

**DOI:** 10.1111/hex.13551

**Published:** 2022-06-28

**Authors:** Anett S. Tarberg, Morten Thronæs, Bodil J. Landstad, Marit Kvangarsnes, Torstein Hole

**Affiliations:** ^1^ Medical Department Møre og Romsdal Hospital Trust Ålesund Norway; ^2^ Department of Clinical and Molecular Medicine, European Palliative Care Centre (PRC), Faculty of Medicine and Health Science Norwegian University of Science and Technology (NTNU) Trondheim Norway; ^3^ Department of Health Sciences in Ålesund, Faculty of Medicine and Health Sciences Norwegian University of Science and Technology Ålesund Norway; ^4^ Cancer Clinic, St. Olav Hospital Trondheim University Hospital Trondheim Norway; ^5^ Department of Health Sciences Mid Sweden University Ostersund Sweden; ^6^ Levanger Hospital Nord‐Trøndelag Hospital Trust Levanger Norway; ^7^ Unit of Research, Education and Development Ostersund Hospital Ostersund Sweden; ^8^ Department of Research and Innovation Fagavdelinga, Møre og Romsdal Hospital Trust Ålesund Norway; ^9^ Faculty of Medicine and Health Sciences Norwegian University of Science and Technology Trondheim Norway

**Keywords:** advance care planning, caregivers, ethical decision‐making, neoplasms, palliative care, patient, physicians

## Abstract

**Introduction:**

Patient participation is essential for quality palliative care, and physicians play a crucial role in promoting participation. This study explores physicians' perceptions of patients and family caregivers' involvement in the different phases of the palliative pathway and employs a qualitative design with thematic analysis and a hermeneutic approach.

**Methods:**

A purposive sampling included physicians who worked in different phases of the palliative pathway. In‐depth, semi‐structured interviews were conducted with 13 physicians in Norway between May and June 2020.

**Results:**

Three main themes illustrate physicians' perceptions of patients' and family caregivers' involvement: (1) beneficence for the patient and the family caregivers in the early phase, (2) autonomy and shared decision‐making in the middle phase, and (3) family involvement in the terminal phase.

**Conclusion:**

The physicians perceived bereavement conversations as essential, particularly if the pathway had been challenging. They also perceived patient participation and family caregivers' involvement as contextual. The results reveal that participation differs across the different phases of the palliative pathway. This type of knowledge should be included in the education of health‐care professionals. Future research should explore elements vital to successful patient participation and family involvement in the different phases of care.

**Patient or Public Contributions:**

Family caregivers were involved in a previous study through individual interviews. The same interview guide used for the family caregivers was used when interviewing the physicians. The family caregivers' contribution led to nuanced questions in the interviews with the physicians, questions leaning on their stories told.

## 1 INTRODUCTION

Patient participation is a core element of patient‐centred palliative care,^1^, ^2^, ^3^, ^4^ and patients are encouraged to participate in decisions about their health care.^5^, ^6^, ^7^ Patient participation and involvement are key elements of good palliative care and follow‐up.^3^, ^8^, ^9^, ^10^ However, unmet needs related to patient participation and family caregivers' involvement have been reported.^3^, ^11^, ^12^ Patient participation begins with health‐care professionals' understanding their patients' preferences and needs for care, creating good relationships and exploring each patient's ability to participate, regardless of their illness and resources.^13^, ^14^, ^15^ Thus, physicians should encourage patients to communicate their values and preferences and allow shared decision‐making to increase their awareness and understanding of treatment options and possible outcomes.^16^, ^17^ However, primary care physicians may face challenges in end‐of‐life care, especially in communication and pain relief^18^ because their roles are not well defined and may vary widely depending on the cases.^19^

This study examined physicians' perspectives on patient participation and family caregivers' involvement in palliative cancer care. The palliative care pathway was divided into three phases. The first phase comprised the initial days following the diagnosis of an incurable disease and began at the point where subsequent treatment was determined to be palliative. The middle phase constituted the time between the early phase and the terminal phase, and the terminal phase comprised the last weeks and days before death.^20^

### 1.1 Background

Life‐threatening illness is difficult for patients.^21^, ^22^, ^23^, ^24^ A focus on symptom relief as well as psychosocial and spiritual aspects are essential elements of palliative care.^7^ Patient‐centred care also strengthens patients' autonomy.^10^

Shared decision‐making can improve patient and family involvement; however, physicians and health‐care personnel may not be aware that participation in decision‐making could be hindered or encouraged based on how they promote options or roles.^8^, ^25^ Physicians must offer patients and family caregivers an opportunity to discuss end‐of‐life issues.^5^ Health‐care providers often do not ask patients whether they want to participate, and patients do not express the kind of roles they want to play in the decision‐making process.^8^, ^25^ According to Tamrisa et al.,^26^ most physicians prefer honest and open communication when discussing patients' concerns and expectations, whereas others choose to adhere to treatment protocols, without explaining the alternatives in the belief that they are giving patients false hope.^27^

Patients who want to be involved and play an active role in the decision‐making process may find it challenging when decisions are delayed and alternative treatment options are not discussed.^1^, ^26^ Several communication gaps have been identified in cancer care, including shared decision‐making, unmet needs, open communication^28^ and the opportunity to be heard without being judged.^29^ Inadequate information provision, lack of practical guidance and insufficient support from health‐care professionals are other challenges.^30^ Essential competencies for patient and family satisfaction include prognostication, conflict mediation, empathic communication and family‐centred care.^9^

Family caregivers play a critical role in the decision‐making process, with patients often taking the final decisions after consulting with their family caregivers.^31^ While patients, their families and health‐care professionals may have different views on prioritizing the different palliative care dimensions,^32^ they need to cooperate to contribute to the patient's wishes and needs.^3^ It is also essential to initiate end‐of‐life care early because delayed communication may lead to missed opportunities.^33^, ^34^

Advance care planning (ACP) and general practitioners' (GPs) involvement have improved palliative care. GPs are central in introducing ACP; simultaneously, GPs have also reported difficulties in introducing ACP when patients are receiving treatment in a hospital.^35^ Introducing ACP can be an autonomous decision, as some patients may not be willing to have that conversation.^36^ Furthermore, family caregivers report a lack of involvement in and preparation for the dying process.^12^ Thus, there is a gap between the guidelines and the emotional and psychological support received in palliative care.^12^, ^33^, ^37^

In all the three phases of palliative care, the patients are primarily at home, which is recommended.^38^, ^39^ In home‐based care, patients and family caregivers rely on GPs and nurses' medical proficiency, availability, person‐focused approach and proactiveness.^13^, ^40^, ^41^ The home‐based care provided by physicians and contracted professionals outside the family could also effectively support home deaths.^42^, ^43^

We believe that greater insight into palliative care participation will be useful in improving care. As physicians play a key role in ensuring quality palliative care and promoting patient and family involvement, we examined how physicians perceived patient participation and family involvement in the different phases of the palliative care pathway.

### 1.2 Ethical principles and theoretical framework

We applied the four principles of biomedical ethics^44^ and the approach of Thompson et al.^45^ to explore patient participation. These ethical principles and the theoretical framework of patient participation were appropriate and were used as theoretical lenses in the analysis.

Four principles of health care that form a moral framework are highlighted^44^: (1) Respect of autonomy: refers to respecting the decision‐making capacity of autonomous persons and their right to participate, ensuring informed consent in important decisions. Therefore, the health legislation's provision on consent competence might be necessary to practice autonomy. This principle obliges disclosing information to probe for and ensure understanding and voluntariness, and to provide adequate decision‐making; (2) Nonmaleficence: refers to protecting against unnecessary harm. Assessment and treatment are burdensome and can involve a health risk. Therefore, the risk of injury should be less than the expected benefit of examinations, treatment, and other healthcare; (3) Beneficence: refers to providing benefits and balancing benefits, burdens and risks. One ought to prevent and remove evil or harm. One ought to perform and promote good. In addition, beneficence balances the utility value and benefits of treatment choices against the risk and strain to which the person is exposed; and (4) Justice: refers to fairness in the distribution of benefits and risks. It is about the management and distribution of opportunities, health benefits and resources. Costs and resources should be distributed in a fair way and managed with the intention to treat cases equally.^44^

We used five levels of involvement that ranged from noninvolvement to full autonomy, based on the framework of Thompson et al.^45^ Participation comprised five components: (1) contributing to action sequences, (2) influencing the problem definition, (3) sharing the reasoning process, (4) influencing decision‐making and (5) experiencing emotional reciprocity. They were in turn based on three core elements: components, levels and context.

A patient's participation depends on the context and may change during their illness. The health‐care provider has a responsibility to promote patient participation through dialogue and information sharing.^44^, ^45^

### 1.3 Research question

This study's research question was: How do physicians perceive patient participation and family caregivers' involvement in the different phases of the palliative pathway?

## 2 METHODS

### 2.1 Design

The study employed a qualitative design using thematic analysis^46^, ^47^ and a hermeneutic approach.^48^, ^49^ Interviews were based on open‐ended questions,^50^ and the consolidated criteria for reporting qualitative research checklist was used to complete the reporting.^51^

### 2.2 Participants

Thirteen Norwegian physicians treating palliative care patients were recruited through purposive sampling.^50^ Both palliative care physicians and GPs treating palliative care patients with cancer were included. The inclusion criteria were physicians with experience in palliative care and treating patients and family caregivers using primary care services. A contact person in health care recruited the physicians. Although 15 physicians were approached to participate, only 13 were accepted. Their demographic characteristics are summarized in Table 1.

**Table 1:** Demographic data

Demographic data	Participants (*N* = 13)
Gender	
Men	10
Women	3
Age (years)	
41–50	7
51–60	4
61–70	2
Workplace	
Hospital	6
Primary care	7
Experience as a physician (years)	
10–15	2
16–20	5
21–25	0
26–30	4
>30	2

### 2.3 Data collection

The interviews took place from April to May 2020 and were conducted by the first author. Owing to the COVID‐19 pandemic, all the interviews were performed individually through video meetings.

An interview guide with open‐ended questions (Table 2) was developed based on the study's aim and previous research.^12^, ^20^, ^50^ The questions focused on how physicians perceived patient participation and family caregivers' involvement in the palliative pathway. The interviews lasted between 35 and 60 min.

**Table 2:** Interview guide

Can you tell me how you experience palliative care?
How are patients and family caregivers involved in the different phases of the pathway?
What is important when communicating with patients and family caregivers in different phases of the palliative pathway?
In your experience, what kind of information is important to communicate?
How do you wish to collaborate with family caregivers throughout the pathway?
What is important about the nature of the care offered in different phases of the palliative pathway?
What challenges and ethical dilemmas did you experience?
Do you want to add something else?

When the interviews produced no new information, the authors discussed the possibility of saturation, and found the data to be rich and dense, and saturated with preliminary themes.^52^, ^53^

### 2.4 Data analysis

The interviews were audio‐recorded and transcribed verbatim by the first author. All the authors read the interview transcriptions to gain a holistic impression of the data.^50^

A thematic analysis and the six steps of Braun and Clark^46^, ^47^ were used to analyse the data. First, all the authors read and reread the transcribed interviews and noted their initial ideas. We also discussed their overall understanding of the data set's coding phases. The first author coded the interviews related to participation in the early, middle and terminal palliative care phases. Second, the authors constructed a coding tree guided by the four ethical principles (i.e., autonomy, nonmaleficence, beneficence and justice)^44^ and the involvement perspective of Thompson et al.^45^ Third, the authors identified key quotations. In the fourth step, the authors discussed subthemes and themes. The analysis was inductive as well as deductive. We worked back and forth between the subthemes and themes until we had established a comprehensive set of themes. Then deductively, we looked back at the data from the themes to determine if more evidence could support each theme. Then the subthemes were abstracted into three main themes, which illustrate physicians' perceptions of participation in the different phases of the palliative pathway. In the fifth step, the authors validated the naming of the themes through communicative validity.^50^ In the last step, the first author wrote down the results, based on feedback from the other authors.

The analyses employed a hermeneutic approach, recognizing the influence of preunderstanding on data interpretation.^49^ We developed a new understanding through group discussions in which all the authors were engaged.^50^ The first author has worked as an oncology nurse in primary care for 10 years. Leaning on a hermeneutic approach, her preunderstanding influenced data interpretation.^49^ The hermeneutic circle conveys the meaning that the parts depend on the whole and the whole depends on the parts.^48^, ^49^ Thus, we developed a deeper understanding of physicians' perceptions of patients and their family caregivers' involvement in the different phases of the palliative pathway. In a hermeneutic approach, the researcher is a participant and producer of knowledge as the data are collected, analysed and interpreted.^49^

### 2.5 Ethical considerations

The project adhered to the guidelines for research ethics laid down by the Declaration of Helsinki. The study was considered by the ethics committee and did not need approval.

All the physicians were given oral and written information on the study and could withdraw at any stage. The first author obtained written informed consent from participants before the interviews. All data were anonymized.

## 3 FINDINGS

Thirteen physicians were interviewed (Table 1). Three themes related to the different phases in the palliative pathway were identified: (1) beneficence for the patient and the family caregivers in the early phase, (2) autonomy and shared decision‐making in the middle phase and (3) family involvement in the terminal phase.

### 3.1 Beneficence for the patient and family caregivers in the early phase

#### 3.1.1 Emotional reciprocity

The physicians described the early phase as demanding for both patients and family caregivers. Patients in this phase were affected by the side effects of treatment, weakened general conditions and loss of roles. Physicians reported that the patients needed to be informed about the transition from curative to palliative treatment options and what they could expect from such options. In this phase, information should meet the patients' and family caregivers' emotional needs: ‘Getting cancer is terrifying and dying is difficult; we all want to live’ (13). The physicians wished that providing information in this phase should contribute positively to the process of preparing for death, listening and creating openness. Those involved in the treatment need to discuss matters with each other to coordinate information with the patient and their family caregivers. The physician expressed a paternalistic attitude; however, they considered it important to establish a close therapeutic relationship with patients and their family caregivers. Ideally, they conveyed that they preferred to give information to patients and caregivers simultaneously.

#### 3.1.2 Physicians' treatment choices

The physicians assumed that patients and family caregivers lacked the required medical knowledge to participate in treatment choices and emphasized their responsibility as physicians: ‘We must understand that it is our responsibility to choose the best treatment. It creates insecurity if the patient has to choose their treatment’ (13). This shows that the physicians were concerned with doing what they thought would benefit the patient and family caregivers.

The physicians considered compassionate care, including information and dialogue essential in the transition from curative to palliative treatment. According to the physicians, this could be a sliding transition, where patients' understanding of their treatment could occasionally be incompatible with professionals' understanding. The physicians acknowledged that they were sometimes unsuccessful in informing patients about the transition from curative to palliative treatment. The physicians expressed that ideally, both patients and family caregivers should be involved in this process.

#### 3.1.3 Creating security for patients and family caregivers

The physicians conveyed that it is important for patients and family caregivers to know who was responsible for the treatment, the physicians in the hospital or the GP: ‘It is important that family caregivers are well informed and included in decisions about who will follow‐up’ (3). To create a sense of security for patients and family caregivers, they emphasized the importance of constructing a palliative care plan. Physicians also told it necessary to communicate the point of contact in case the illness worsened, or other potential medical challenges were encountered.

The physicians were also concerned about ethical dilemmas associated with providing information. They considered the needs of patients and family caregivers, which had to be adapted to patients' health literacy as essential. The physicians also saw it necessary to provide individualized information. Information and dialogue with family caregivers were seen as essential for planning a good course of treatment. The physicians also noted potential challenges in predicting family caregivers' care resources in the palliative care process.

The physicians emphasized their duty of confidentiality towards the patient. It was vital that the patient decided how and to what extent family caregivers could be involved. Some patients did not want the information to be passed on to their family caregivers. This might be an ethical dilemma for the physicians. Some of them said that they urged patients to inform family caregivers based on their best interests.

### 3.2 Autonomy and shared decision‐making in the middle phase

#### 3.2.1 Patient and family caregivers' involvement

According to the physicians, the middle phase could be a comparatively calmer period in which the patient and family caregivers prepare for the death. Patient autonomy was considered particularly important: ‘It is the patients who own this process’ (8). The physicians considered that ACP was a good tool. It was essential to have conversations with the patient and family caregivers about the pathway, their future expectations and their thoughts regarding participation. The physicians highlighted challenges in meeting patients' and family caregivers' differing needs for information and involvement in the treatment and emphasized the need to be open about the disease's progression and include patients and family caregivers in discussions about possible future challenges and choices that would have to be made: ‘I experience that most people prefer to have an open and good dialogue. They are grateful after the difficult conversation’ (12). In the interviews, there was a lack of reflections on how patients and family caregivers experienced ACP.

#### 3.2.2 Continuity of care

The physicians emphasized that building trust was important. One GP explained how he actively worked to create trust and security by having routine consultations with patients: ‘I think being assigned appointments regularly makes it easier for the patient, and they do not feel that they are taking my time. I am the one who gives the time. If they do not want the consultation, they actively cancel’ (8).

The physicians felt it would be easier to outline responsibilities and create security if patients did not constantly have to deal with new health‐care personnel in the hospital and the municipality. Information could be overlooked if there was not enough confidentiality around crucial conversations.

The physicians working in palliative teams emphasized the importance of working in multidisciplinary teams. They highlighted that the nurses often had an important role in coordinating the care and the treatment.

#### 3.2.3 Family caregivers as resources

The physicians highlighted family caregivers as a crucial resource in palliative care and emphasized the need to spend time with them. The closer the patient was to the terminal phase, the greater the need to cooperate with family caregivers. Close cooperation was also crucial when death at home was planned: ‘Family caregivers must be in place. It is so easy and so difficult at the same time’ (7).

Security, accessibility, information and planning for the time ahead were viewed as essential components of care. Physicians cited examples of family caregivers who mobilized help to ensure that the patient was adequately cared for, as providing care entailed considerable responsibility. As family caregivers have different levels of resources, physicians were often concerned about the adequacy of resources, although family caregivers often mobilized more resources than they expected.

### 3.3 Family involvement in the terminal phase

#### 3.3.1 Early involvement of family caregivers

The physicians highlighted how, in the terminal phase, they tried to support family caregivers emotionally and identify common perspectives and solutions. Family caregivers' involvement in the palliative pathway was akin to living the grieving process: ‘The most important thing we can do to help them cope with their grief is what we do along the way. If we did a bad job, a bereavement conversation will not save the grieving process’ (9). Family caregivers should feel validated, heard and respected throughout. In this process of understanding, having a plan for what may lie ahead was an essential issue.

Some physicians said they had become better at involving the family caregivers, emphasizing that this has led to people increasingly declining a bereavement conversation. One of the physicians said he could tell who would need a bereavement conversation based on how stressful the palliative pathway was and whether the family caregivers considered the death as traumatic. Providing information to the family caregivers about how they could experience the time after their family member's death was essential in supporting the mourning process.

#### 3.3.2 Autonomy maintained by family

In the terminal phase, the family often maintains the patient's autonomy. The physicians depend on the information provided by family caregivers to consider the patient's interests. Physicians said it was essential to clarify with the patient, early in the pathway, that the physicians would contact family caregivers when the patient was tired or otherwise indisposed.

Family caregivers were described as a link between the patient and the physicians; thus, a good relationship with family caregivers was vital: ‘We used to support the family caregivers and help them understand. Sometimes the symptoms bother the family caregivers more than they do the patient’ (2). Additionally, regular follow‐ups and a continuous flow of information provision were necessary to satisfy family caregivers' concerns. Some patients may find relief in letting family caregivers play a more prominent role. Physicians also noted family caregivers' fear of not being able to adapt to progressive disease symptoms and not being able to cope with a worsening situation. The terminal phase could be challenging regarding symptom relief and the level of care required from family caregivers.

#### 3.3.3 Bereavement conversations

The physicians said conversations with the bereaved after the patient's death were important for processing the challenging experiences during the pathway: ‘If there had been complicated processes and stress about treatment clarifications regarding symptom relief, the physicians might be involved in the bereavement conversation’ (12). A bereavement conversation could help summarize the challenging events and provide answers to questions that had remained unanswered. It was also an opportunity to discuss possible feelings of guilt. They highlighted the importance of bereavement conversations to avoid lifelong trauma; half an hour of bereavement conversation could prevent the bereaved from developing dark thoughts for the rest of their lives.

The physicians viewed bereavement conversations as positive and were often conducted in a friendly atmosphere. They experienced that the bereaved often seemed lighter in spirit after such conversations.

## 4 DISCUSSION

In this study, patient participation and family caregivers' involvement has been studied from the perspective of physicians. The interviews gave rich and thick descriptions of physicians' perceptions of patient and family caregivers' involvement in the different phases of the palliative pathway. The result might be interpreted as the physicians expressed that the ethical principle of beneficence characterized the first phase. The principle of autonomy and shared decision‐making characterized the middle phase. Family involvement was considered crucial in the terminal phase. This study offers new insight into physicians' perceptions of patient participation and family caregivers' involvement in the different phases of the palliative pathway.

The physicians perceived an ethical dilemma between beneficence and patient autonomy. They saw the importance of balancing the burden and risks for the patient and the family.^44^ The physicians considered it their responsibility to choose the best treatment for the patient, this might be seen as an ethical dilemma in relation to safeguarding the patient's autonomy.

It is clear from the physicians' accounts that finding a balance between the different ethical principles^44^ is a process that evolves over time and requires competence and practical experience in the field of palliative care. The principle of autonomy emerges as a common thread that runs through the entire palliative process, modified by the principle of beneficence, especially in the early stage of the palliative pathway and in the involvement of family caregivers. Physicians balance the two principles of beneficence and autonomy, especially information and communication in the early phase. Balancing communication within participation depends on how much patients can and want to participate and the context,^44^, ^45^ as well as their individual preferences.^26^ The physicians also discussed family caregivers' involvement and the balance between autonomy and the principle of nonmaleficence. Family caregivers often have a say in autonomous decisions^31^ even when their needs or wishes differ from those of the patient.^32^

Extant research has indicated the importance of the coordination and integration of care and information and communication as primary goals. Emotional support and the involvement of the family are vital.^3^, ^8^, ^10^ The physicians expressed that beneficence was important for the patient and the family caregivers early in the pathway.

We found differences among physicians in how they viewed the decision‐making process. Sometimes they prioritized autonomy and encouraged a high degree of patient involvement, while at times, they had a mildly paternalistic attitude. Thompson et al.^45^ demonstrated how involvement differs in terms of context, levels and components. In our findings, physicians made some treatment choices to avoid unnecessary risks to the patient. This is consistent with previous findings.^16^, ^44^ The physicians in our study highlighted patient autonomy, especially in the middle phase, although this could conflict with the ethical perspective of beneficence to the family caregivers.^44^ Extant studies found that patient‐centred care could be at the expense of family caregivers, who tend to be neglected.^12^, ^32^, ^37^

We found that physicians are aware of the significance of involving the patients and family caregivers. In contrast, prior studies have shown that physicians do not meet the patient's and family caregivers' information needs.^1^, ^26^, ^28^, ^29^ The physicians in our study also discussed a shift in autonomy from the patient to the family caregivers, in which they played an active role in helping patients hand over the authority to make choices to the family caregivers—doing good to the patient was the reason for this initiative.^44^ Previous research has also indicated limited involvement of family caregivers and a lack of preparation for the terminal phase.^12^, ^33^ This lack of participation does not correspond with our results; indeed, experienced physicians acknowledged the importance of their involvement throughout the pathway. In the mentioned studies, however, it is a clear finding that physicians and family caregivers emphasize the importance of cooperation and involvement in the first phase.^12^

Our study confirms ACP's significance, which includes patients and family caregivers in planning palliative care. Many of the physicians highlighted the importance of formulating a plan to ensure safety and predictability for the patient and family caregivers, which is consistent with earlier research emphasizing ACP's importance early on in the pathway to promote predictability.^3^, ^20^, ^35^ Research shows that the concept of quality in palliative care has to be familiar to patients, family caregivers and health personnel,^32^, ^35^ and highlight patients and caregivers' unmet needs, especially regarding communication with health‐care professionals.^28^, ^37^ The physicians in our study mentioned the importance of building trust with both the patient and the family caregivers to include them in discussions about the future and formulate plans.

They considered family caregivers as a resource for the patient throughout the pathway. This finding is consistent with Lamore et al.^31^ who revealed the essential role of family caregivers in the final decision‐making process. Family caregivers' early involvement in the pathway was also highlighted in the Lancet Oncology Commission.^3^ The physicians noted that involving family caregivers early in the palliative care pathway and ensuring that they closely monitored the process led to better grief processing. Many believed that the need for bereavement conversations had diminished, reflecting an increase in family caregivers' involvement and adaptation.

### 4.1 Strengths and limitations

One of the strengths of this study is the physicians' long‐term experience in palliative care. The physicians were both men and women of various ages, worked in primary and specialist care, and were from both rural communities and larger cities, and from local and regional hospitals. Gender analyses were not in focus in the study. Men and women participated in the study because we wanted variation and, our theoretical perspectives do not focus on gender. Although this study was conducted in Norway, the findings may be transferable to other countries with similar health‐care environments.^54^

A limitation might be that the first author has worked as an oncological nurse in primary care for 10 years, and has a preunderstanding. However, all the authors collaborated in the interpretation and development of a shared understanding of the data, ensuring a holistic perspective.^49^, ^50^, ^54^ Additionally, the theoretical framework^44^, ^45^ strengthened the transparency of the interpretation.^54^

Observations in addition to interviews could have been applied to collect data.^45^ Method triangulation in further research might be valuable to develop a more comprehensive, consistent and coherent understanding of how patient participation and family involvement occurs in practice.

### 4.2 Implications

This study provides insight into the complex concept of participation and the four ethical principles: autonomy, beneficence, nonmaleficence and justice. The dilemma expressed by physicians between ethical principles and encouraging patient participation and family caregivers' involvement can be transferable to patients with incurable diseases. The results reveal a need for physicians to see participation as a contextual process, which should be a topic in further specialist and medical education. In addition, future studies should determine the factors that are essential to successful patient participation and family involvement in palliative care. Future research should give more attention to the way doctor–patient communication is incorporated into the multidisciplinary palliative care plan.

The physicians in this study involved the family caregivers early and throughout the palliative pathway; this should be highlighted in health personal education and future research. In addition, the conflict in balancing ethical principles and the consequences for clinical work should be highlighted, both in daily practice and in further research. In the future, investigation of patients and family caregivers, as well as nurses' and policymakers' perspectives on participation, involvement and ethical principles, could present a more holistic understanding for all, including researchers and other stakeholders.

## 5 CONCLUSION

The physicians perceived that patients' participation and family caregivers' involvement differ across the various phases of the palliative pathway. The ethical principle of beneficence for patient and family caregivers is seen as most important in the first phase. In the second phase, the physicians saw autonomy and shared decision‐making as crucial. In the terminal phase, the physicians perceived family involvement as essential. The physicians were concerned with patient participation and family involvement throughout the palliative pathway. The study showed that the physicians perceived patient participation and family caregivers' involvement as contextual and that participation differs across the different phases of the palliative pathway.

## AUTHOR CONTRIBUTIONS

Anett Skorpen Tarberg, Torstein Hole, Morten Thronæs, Marit Kvangarsnes and Bodli Landstad designed the study. Anett Skorpen Tarberg collected the data. All authors contributed to the analysis and interpretation of data. Anett Skorpen Tarberg and Torstein Hole drafted the manuscript. All authors have contributed to revising this article critically and contributing with important intellectual content. All authors agreed to be accountable for all aspects of the work.

## CONFLICT OF INTEREST

The authors declare no conflict of interest.

## Data Availability

The data that support the findings of this study are available on request from the corresponding author. The data are not publicly available due to privacy or ethical restrictions.
